# Current Insights Into the Role of Neuropeptide Y in Skin Physiology and Pathology

**DOI:** 10.3389/fendo.2022.838434

**Published:** 2022-03-28

**Authors:** Zoya T. Anderson, Alex D. Dawson, Andrzej T. Slominski, Melissa L. Harris

**Affiliations:** ^1^ Department of Biology, University of Alabama at Birmingham, Birmingham, AL, United States; ^2^ Department of Dermatology, Comprehensive Cancer Center, Cancer Chemoprevention Program, University of Alabama at Birmingham, Birmingham, AL, United States; ^3^ Veteran Administration Medical Center, Birmingham, AL, United States

**Keywords:** neuropeptide Y, skin, physiology, pathology, stress

## Abstract

Neuropeptide Y is widely distributed within the body and has long been implicated as a contributor to skin disease based on the correlative clinical data. However, until recently, there have been few empirical investigations to determine whether NPY has a pathophysiological role in the skin. Due to appearance-altering phenotypes of atopic dermatitis, psoriasis, and vitiligo, those suffering from these diseases often face multiple forms of negative social attention. This often results in psychological stress, which has been shown to exacerbate inflammatory skin diseases – creating a vicious cycle that perpetuates disease. This has been shown to drive severe depression, which has resulted in suicidal ideation being a comorbidity of these diseases. Herein, we review what is currently known about the associations of NPY with skin diseases and stress. We also review and provide educated guessing what the effects NPY can have in the skin. Inflammatory skin diseases can affect physical appearance to have significant, negative impacts on quality of life. No cure exists for these conditions, highlighting the need for identification of novel proteins/neuropetides, like NPY, that can be targeted therapeutically. This review sets the stage for future investigations into the role of NPY in skin biology and pathology to stimulate research on therapeutic targeting NPY signaling in order to combat inflammatory skin diseases.

## Introduction

Neuropeptide Y (NPY) is a small 36 amino acid peptide that is well recognized as a common neuropeptide produced within the hypothalamus of the brain (1, 2). In the central nervous system, it is mainly synthesized by neurons of the sympathetic system (2), or in different parts of the brain including hypothalamus, hippocampus (predominantly arcuate nucleus and dentate gyrus), where it plays important roles in the regulation of feeding behavior, storage of energy (3, 4), stress and anxiety responses (5–7), and affecting blood pressure, nociception, and circadian rhythm (8, 9). These actions are mediated by interaction with five, G protein-coupled, membrane-bound, NPY Y receptors (Y1R, Y2R, Y4R, Y5R, Y6R) (2, 10–13). On the central level, NPY is part of important neuroendocrine loops involving corticotropin releasing hormone (CRH), proopiomelanocortin (POMC)-derived peptides, and other neuropeptide signaling systems.

In the years since its discovery, NPY has been shown to be the most widely distributed neuropeptide throughout multiple tissues in a variety of organisms (2, 12, 13). There is evidence of NPY expression in almost every tissue (2, 10, 14, 15), with its expression and synthesis being constitutive or inducible in nearly every type of cell (16–22). Due to its wide distribution, NPY has pleiotropic roles throughout the body in the central biological processes mentioned above, as well as in the periphery by regulating cell proliferation (23, 24), immune responses (25–27), and vasoconstriction (28–30). Despite its known functions in several physiological and pathological processes, the exact effects that NPY has on individual tissues remains poorly understood. It is clear that the specific effects of NPY are dependent on the cell type(s) and the receptor(s) involved (2, 11, 31, 32), making rote assumptions on NPY function potentially misleading when comparing across tissues.

The skin, together with its adjacent adipose tissue that comprises the hypodermis, represents the largest body organ and is continuously exposed to different chemical, biological and physical stressors (33, 34). In addition to its important barrier functions (35), the skin has immuno- and thermoregulatory functions (36, 37), can affect body homeostasis (38), and communicates with the brain in a sophisticated manner through ascending nerves and humoral signals entering circulation (39–43). The integumental homeostasis is regulated by a local neuroendocrine system in coordination with immune and pigmentary systems, which uses the same neuro-mediators and regulatory loops as those operating in the brain, endocrine (38, 44, 45), and immune (37) systems. This system, in response to different noxious factors, can send signals to central coordinating centers to counteract the stressors and restore local, or affect global, homeostasis (34, 35, 43, 46). Importantly, the skin expresses all of the neuroendocrine elements that interact with NPY in the brain, such as local cutaneous CRH and POMC systems (44, 47–49).

With these complex processes in mind, it may be no surprise that the role(s) of NPY in the skin currently remain a mystery to scientists and dermatologists alike. There has been evidence to suggest genetic associations of NPY with vitiligo as well as evidence that shows differential expression of NPY in these and other skin pathologies. These data, while merely correlative, support the long-standing hypothesis that NPY can have pathomechanistic roles in the skin (38). To better understand mechanisms by which NPY contributes, or might contribute, to skin physiology or pathology, the field would benefit from a succinct review of the various ways in which NPY has been associated with skin disease and how NPY can affect the various cell populations in the skin.

In addition to NPY, the family of peptides encompassing NPY contains two other peptides, known as PYY and PP. The first to be discovered in this family was PP, discovered in 1968, while NPY and PYY were isolated in 1982 (50, 51). Originally, both hormones were discovered to have major roles in the gut-brain axis, with NPY being expressed throughout the gut-brain pathway, though preferentially in neurons, whereas PYY and PP is expressed most abundantly in the lower gastrointestinal tract (52, 53). More recently, the expression of the NPY family of peptides and their receptors has been described in the skin, opening up new avenues of investigation outside of the gut-brain axis (28, 30, 54). In addition to the many functions of NPY in the skin described in this review, it should also be noted that functions of PYY outside of the gut-brain axis have also been described (55). One such function described in the skin is the activity of both PYY and NPY as antibiotic agents, with both PYY and NPY being shown to degrade bacterial membranes to irreversibly inhibit bacterial proliferation (56). While the importance of all three peptides cannot be overlooked, this review will be focusing mainly on the presence of NPY in the skin and its role in skin pathology. In this review, we will discuss what is currently known about the associations of NPY with various skin pathologies and discuss the potential mechanisms by which NPY is involved in these disease processes.

## Expression of NPY Protein in Skin Pathology

In healthy human skin, NPY is detectable around the blood vessels, nerve fibers, sweat glands, subcutaneous adipocytes, basal epidermal cells, and cells of the hair follicle’s outer root sheath (38, 57–63). NPY is also present at low levels in the circulation of healthy humans (63–66). In addition to the physiological expression pattern of NPY, several groups have shown that NPY is elevated in the affected skin and/or circulation of humans suffering from various skin pathologies, including atopic dermatitis, cutaneous melanoma, psoriasis, and vitiligo.

### Atopic Dermatitis (AD)

Atopic dermatitis (AD) is an inflammatory disease in which itchy rashes appear on the skin due to various irritation-inducing factors (67). This is a common and chronic disease with multiple etiological influences, including those of immunological, genetic, and psychological origins (64). There is no cure for AD, and it can have detrimental impacts on a person’s quality of life *via* inducing social stigma and negatively affecting psychological well-being.

Generally, NPY expression has been shown to be elevated in the lesional skin of AD patients relative to healthy volunteers. Oh et al. found significantly more NPY-expressing cells and NPY-like immunoreactivity (NPY-IR) in the epidermis of lesional AD skin compared to the skin of healthy volunteers, along with a significant association between NPY expression in lesional AD skin and psychological stress, as well as pruritis, or itchiness (68). Pincelli and colleagues found that the lesional skin of several AD patients showed the presence of Langerhans cells, a specialized dendritic cell found in the epidermis of the skin, with NPY-IR, which was not seen in the skin of healthy volunteers (61, 69). These studies suggest that the source of NPY in these cases may be local to cells of the epidermis rather than nerve derived. This is further supported by the fact that Tobin and colleagues report no notable change in the density of NPY-expressing nerve fibers within the dermis of lesional skin from AD patients (70).

Circulating NPY is also increased in AD patients. Salomon and Baran found that NPY is significantly elevated in the plasma of AD patients when compared to that of healthy volunteers (**Table 1**) (66). Additionally, within AD patients, plasma NPY levels were found to be significantly higher in those patients in disease remission compared to patients with active disease, a finding that the investigators hypothesized is due to a selective uptake of NPY by active, lesional AD skin. Salomon and Baran’s observation that NPY is significantly elevated in the plasma of AD patients was further supported by Hodeib and colleagues, who found that plasma NPY is significantly elevated in AD patients with moderate and severe disease compared to patients with mild AD and healthy volunteers (**Table 1**) (64). The exact source of NPY during this disease is an important, yet still unanswered, question. Investigators have hypothesized that the NPY that is present in the circulation and lesional skin of AD patients is released both centrally and locally from the skin’s nerve endings (64, 66). As this hypothesis has yet to be evaluated, elucidation of the source of NPY, as well as the contributions that this peptide has in AD disease, will determine whether and how this peptide may be targeted in the treatment of AD.

**Table 1:** The concentrations of NPY in the circulations of healthy volunteers and patients with skin pathologies.

Skin Pathology (tissue)	Healthy Volunteers (pg/mL)	Patients (pg/mL)	Disease Classification	Reference
Atopic Dermatitis (plasma)	35.51 ± 97.71	54.59 ± 39.63*	Active	66
		66.75 ± 39.98*	Remission	
	11.61 ± 7.4	34.7 ± 15	Mild	64
		43.9 ± 9.8*	Moderate	
		72.9 ± 11.9*	Severe	
Psoriasis (serum)	455.5 ± 154	493.9 ± 183*	Overall	65
Vitiligo (plasma)	130.4 ± 62.6	209.1 ± 60.5**	Overall	63
		177.2 ± 59.9*	Local	
		235.2 ± 54.2**	Generalized	
		199.3 ± 45.3**	Segmental	

NPY is significantly elevated in the circulation of patients with AD and vitiligo, but not psoriasis. Data are shown as mean ± standard deviation. *p < 0.05. **p < 0.001.

The observations of elevated levels of circulating NPY, along with increased NPY-IR in lesional skin of AD patients, suggest that NPY may serve a pathomechanistic role in the initiation and/or progression of AD. A number of molecular models for AD exist, namely the ‘outside-in’ model that is characterized by the defective expression of fillagrin by keratinocytes leading to a subsequent defective skin permeability barrier, and the ‘inside-out’ model that is characterized by changes in immune cell function, namely an overactive Th2 response associated with increased levels of autoreactive IgEs [reviewed in (71, 72)]. While no direct mechanistic connection between NPY and AD has been established, NPY can regulate cells and processes that intersect with both models for AD. For instance, NPY is a known immunomodulatory factor and is both sufficient and necessary to drive Th2 responses (25, 73, 74). Changes in microbial diversity, along with increased colonization by *Staphylococcus aureus* are also clearly associated with AD severity and disease flares (75–77). NPY has been suggested to participate in shaping the microbiota, albeit in the gut, through both direct antimicrobial actions and indirect effects on regulating innate and adaptive immune responses and similar connections between skin and its microbiota are emerging in dermatology (78, 79). These and additional biological processes in which NPY participates and may overlap with AD pathogenic characteristics are highlighted in Section 4, below.

### Cutaneous Melanoma

Melanoma is a form of cancer in which the pigment-producing cells, called melanocytes, undergo malignant transformation (80). Cutaneous melanoma affects a large segment of the population with high incidence and mortality rates in comparison to other cancers and is the deadliest skin malignancy. While the major etiological factors include solar radiation and genetic background (81–84), different hormonal factors can affect melanomagenesis and progression of melanoma. For example, NPY expression is associated with cutaneous melanoma. Initial reports of NPY expression in primary cutaneous melanomas found that NPY is lowly expressed in melanocytic nevi and highly expressed within the cytoplasm by many types of melanoma tumors (85). In a sample size of 49 primary tumors, Gilaberte and colleagues also reported high NPY expression in primary melanomas which were associated with higher probability of metastasis, such as vertical growth phase with low or no tumor lymphocytic infiltration response. There was also a slight positive correlation between NPY expression and tumor cell proliferation. In a follow-up study, Perez Tato and colleagues evaluated 79 primary tumors and found that superficial spreading melanoma and lentigo maligna exhibit significantly higher levels of NPY expression in comparison to nodular melanoma (86). However, in contrast to Gilaberte et al. these authors report low NPY expression associated with high proliferation, increased metastasis, high peritumoral mast cell density, and reduced patient survival, but no relationship between NPY expression and intratumoral lymphocyte infiltration.

Clearly, these studies are contradictory. Gilaberte and colleagues concluded that NPY may have a pro-tumorigenic role in cutaneous melanoma, and thus its high expression is an indication of poor prognosis for patients. Perez Tato and colleagues instead concluded that high NPY expression is likely an indication of positive clinical outcomes, which they attributed to their larger sample size compared to the Gilaberte study. In relationship to both studies, the source of NPY under these circumstances is unclear, however, previous gene expression analysis detected no significant changes in NPY gene expression levels between normal skin, benign skin nevi, and malignant melanoma (87)[retrieved from **GSE3189**], suggesting an extra-tumoral source. Interestingly, in mice injected subcutaneously with B16-F10 mouse-derived melanoma cells, sympathectomy to reduce sympathetic neurotransmitters (like NPY and noradrenaline) prior to tumor initiation greatly attenuates melanoma growth and yields smaller tumors (88). However, in the same mice, sympathectomy also induces upregulation of NPY in tumor tissue making it unclear as to whether the absence of sympathetic NPY, the induction of tumor NPY, or another sympathetic neurotransmitter contributes to the anti-tumorigenic effect. Evaluating the mechanistic role of NPY in cutaneous melanoma will determine whether this peptide may contribute or prevent disease and will indicate how this peptide could be targeted or used to combat disease pathogenesis and/or progression.

### Psoriasis

Psoriasis is a chronic, inflammatory skin disease in which hyperproliferation of skin cells causes the formation of itchy and scaly dry patches on the skin that was described in modern scientific literature as early as the 1800s (89, 90). Early historical accounts paint psoriasis as an incurable mystery, and that no population demographic or area of the skin appears exempt from it (89). Over a century later, our understanding of psoriasis has changed dramatically, though we are still making new discoveries on the mechanisms that underly psoriasis pathology, such as the resident memory T cells remaining in the skin after psoriasis treatment (91, 92). Like AD, psoriasis is a multi-factorial disease that can have detrimental effects on a person’s psychological well-being and overall quality of life. The expression of NPY in psoriatic skin has yet to be evaluated. However, NPY levels in the serum of patients with psoriasis are not different from those of healthy volunteers (**Table 1**) (65). Despite the lack of as association between NPY levels in circulation and disease status, decreased NPY levels are observed in the plasma of psoriatic patients with pruritis in comparison to those without pruritis (93), and activation of NPY signaling intrathecally can attenuate both mechanical and histaminergic itch in mice (94). Future studies to determine NPY expression in psoriatic skin relative to uninvolved skin, as well as skin from healthy volunteers, will establish whether NPY may be involved in local psoriasis pathology. It must be noted that this involvement may be complex, since other neuropeptides that interact with NPY signaling on the central level, such as CRH and POMC-derived peptides, can affect presentation of inflammatory diseases depending on signaling context (45, 46, 95).

### Vitiligo

Vitiligo is a chronic skin disease that is characterized by progressing skin depigmentation due to the loss or diminished function of epidermal melanocytes. Vitiligo has multiple etiological factors, including those of genetic, autoimmune, neuroendocrine, and oxidative stress origins (96–99). Like other skin diseases, NPY has been found to be elevated in the lesional depigmented skin, as well as in the circulation, of vitiligo patients. Lazarova and colleagues found that in lesional and marginal vitiliginous skin, NPY-IR is elevated when compared to the skin of healthy volunteers. In this vitiliginous skin, NPY was found to be localized around the blood vessels and in the dermis (60). Furthermore, Tu and colleagues quantified the concentration of NPY in tissue fluids isolated from lesional and uninvolved skin of vitiligo patients (63). In general, NPY is significantly elevated in lesional skin when compared to uninvolved skin (**Table 2**). However, this difference is lost in the generalized type of vitiligo, which was hypothesized to be because larger areas of the skin are affected in this type of the disease.

**Table 2:** The concentrations of NPY in skin fluids of vitiligo patients.

Disease Classification	Uninvolved Skin (pg/mL)	Lesional Skin (pg/mL)
Overall	270 ± 87.6	311 ± 55*
Local	230.3 ± 57.7	270.4 ± 39.1*
Generalized	356.2 ± 29.5	366.5 ± 35.9
Segmental	231.5 ± 97.3	308.2 ± 37.4*

NPY levels are significantly greater in affected skin of vitiligo patients compared to unaffected skin. Information from 63. Data are shown as mean ± standard deviation. *p < 0.05.

Tu and colleagues also showed that NPY is elevated in the plasma of vitiligo patients compared to that of healthy volunteers (**Table 1**). Interestingly, they found that NPY is highest in patients with the generalized type of vitiligo, which could also be attributed to the larger areas of skin affected. Because this group found that the concentration of NPY in the skin was consistently higher than its concentration in plasma, they posited that the NPY found in the skin is likely produced and secreted locally from the peripheral nerve fibers in the skin, and that the NPY in the plasma of vitiligo patients is likely spillover from excess peptide in the skin. The role that NPY plays in vitiligo pathology has yet to be uncovered, but Tu and colleagues hypothesized that NPY may influence melanocyte destruction both directly, *via* direct contact between melanocytes and intraepidermal nerve endings, and indirectly, *via* inducing immune cell activation and cytokine production (63). In support of the latter and not the former ideas, Toyoda et al. demonstrated that NPY can cause ‘degenerative changes’ in melanocytes when supplemented in the growth media of organ cultured human skin but does not affect melanocyte morphology or melanin synthesis directly in cultured melanocytes (100). Further, premature and progressive hair graying due to melanocyte stem cell loss is observed in a mouse model of chronic NPY overexpression, although the mechanism of why this loss occurs is unclear (101).

## Genetic Associations of NPY With Skin Pathology

### Vitiligo

Gene association studies have identified polymorphisms in the *NPY* gene as a risk factor for vitiligo in multiple populations. Laddha and colleagues have associated single nucleotide polymorphisms (SNPs) in the promoter region (-399T/C; rs16147) and second exon (+1128T/C; rs16139) of NPY with increased susceptibility for vitiligo in Indian populations (102). In this case, the TC haplotype (+1128 T/-399 C) is associated with a 2.3-fold greater odds for developing vitiligo. Similarly, in an Egyptian population, the C/C and T/C genotypes of the -399T/C SNP are associated with a 3.75- and 4.6-fold increased odds for disease (103). Both the -399 and +1128 SNPs also appear to have functional consequences on NPY gene regulation. Individuals with C/C or T/C genotypes at the -399T/C SNP exhibit elevated NPY gene and protein expression in the anterior cingulate cortex of human post-mortem brain (104). Those with the Leu7/Pro7 peptide sequence (as a consequence of the +1128T/C SNP) exhibit elevated NPY protein in plasma (105). Within neurons, the +1128T/C SNP also increases the secretion of NPY post-translationally (100, 106).These genetic associations of *NPY*, along with the clinical data that show elevated NPY in the plasma and affected skin of some vitiligo patients (63), implicate NPY as a contributing factor to vitiligo pathology and a potential target for therapeutic development in patients with these mutations.

### Cutaneous Melanoma

Evaluation of genomic cancer data available in cBioPortal (http://www.cbioportal.org/) indicates that *NPY* is not frequently amplified (1.12%; 5/447 patients) or mutated (1.34%; 6/447 patients) in the tumors of patients with skin cutaneous melanoma (TGCA Research Network) (107, 108). Neither has GWAS analysis of cutaneous melanoma detected *NPY* as a susceptibility locus (109). Nevertheless, in a study in which copy number variations of neuropeptide and receptor genes were investigated for multiple cancers, the expression of *NPY* and its receptors, *Y1R*, *Y2R*, and *Y5R*, were given negative prognostic Z-scores in cutaneous melanoma (110). Having negative prognostic scores in this study indicates that the expression of NPY and its receptors in cutaneous melanoma is associated with favorable survival outcomes for patients, which is in agreement with the findings from Pérez Tato and colleagues’ clinical study of NPY expression in primary cutaneous melanomas (86).

### Atopic Dermatitis and Psoriasis

To the authors’ knowledge, no genetic associations of NPY have yet been reported for atopic dermatitis or psoriasis.

## A Link Between Stress and Cutaneous Upregulation of NPY

In the skin diseases mentioned above, elevations in NPY protein expression are observed locally within diseased skin. The source of NPY in this context is unknown, however, there is strong evidence indicating that physiological stimulation or psychological stress can lead to cutaneous NPY release from sympathetic nerves or NPY upregulation directly by specific skin cells.

It is well-established that NPY can be released from cutaneous sympathetic nerves to regulate skin vasoconstriction in response to physiological stress, thus making sympathetic nerves a logical candidate to serve as a primary source for NPY in stress-related skin diseases. In human skin, NPY protein expression is, in part, localized to a network-like mesh of adrenergic nerve fibers surrounding arteriole beds (57, 58, 62). The stress of whole-body cooling in humans increases cutaneous blood pressure, and this increase can be blocked by preventing sympathetic nerve transmitter release from nerve endings using bretylium or, more specifically, by localized intradermal administration of BIBP-3226, a selective antagonist for Y1R (111, 112). Interestingly, NPY release is not observed when skin is cooled locally, suggesting that NPY-induced cutaneous vasoconstriction is only involved in the response to the more extreme, systemic cold stress (113).

NPY’s participation in strong sympathetic activation is also observed during exercise-induced stress (114). Plasma NPY levels increase significantly after strenuous (cycling) but not weak (handgrip) exercise, and NPY levels show a positive association with relative perceived exertion. Notably, NPY levels in the plasma after exercise are only one-tenth the level required to produce slight vasoconstriction in human blood vessels *in vitro*. Further, plasma NPY levels correlate closely with increases in the sympathetic co-neurotransmitter norepinephrine rather than the adrenomedullary hormone adrenaline. These observations insinuate, similar to that postulated above for vitiligo, that the primary source of plasma NPY in response to stress is not the adrenal gland, but instead the result of high sympathetic nerve activation localized to target tissues that overflows into the circulation (114, 115).

Systemic elevation of NPY (as measured in plasma) in response to extreme physical or psychological stress is very well-documented (110, 115–119), yet the exact source(s) of NPY in these contexts is rarely identified. Flare-ups in skin diseases are often associated with stressful life events (69, 120–122), and in skin diseases where both local and systemic NPY has been measured, NPY levels are higher in skin than in plasma (**Table 2**) (63). Altogether, these observations support the supposition that stress-related sympathetic stimulation may be a direct source of local NPY elevation observed in some skin pathologies.

In humans, NPY-IR is not limited to sympathetic nerve fibers. As mentioned previously, NPY is detectable in the cells within multiple sites of healthy human skin. Endogenous expression of NPY by these cells suggests non-neuronal sources of NPY could contribute to stress-related skin pathologies *in vivo* and is intuited from non-skin studies. For example, the *NPY* gene is constitutively expressed at a low level in peripheral blood mononuclear cells and in several immune cell populations, including monocytes, macrophages, and T and B lymphocytes, and is upregulated upon their activation (20). Autocrine NPY signaling promotes the ability of mouse peritoneal macrophages to produce normal levels of proinflammatory cytokines after activation (26, 27). Extending this example, upregulation of *Npy* gene expression in subcutaneous fat depots can be induced in mice with exposure to chronic stress in combination with a high fat and high sugar diet through a glucocorticoid-dependent mechanism (123). In this context, fat tissue exhibits significant macrophage infiltration, and it appears that activated macrophages may be the actual source of NPY in fat tissue under the conditions of dietary obesity (22). Conversely, it has also been shown that adipocytes derived from human subcutaneous fat can produce NPY autonomously and can be stimulated to secrete NPY with insulin treatment (59, 124, 125). Independent of the source, NPY induces adipogenesis and release of the adipokines leptin and resistin in preadipocytes *in vitro* and angiogenesis and expansion of fat tissue *in vivo* (123).

The relevance of NPY signaling to skin disease in relationship to adipocytes and obesity is particularly highlighted in psoriasis. Adiposity and weight gain are risk factors associated with psoriasis development, particularly in children (126–128). Increases in leptin and resistin, both proinflammatory adipokines that increase with obesity, and a reduction in adiponectin, an anti-inflammatory adipokine that decreases with obesity, contribute to an inflammatory skin environment [reviewed in (129)]. One key player in this inflammation is thought to be adipose tissue macrophages, which in human subcutaneous fat, significantly increase in abundance in correlation with body mass index or adipocyte size (130). Inflammatory cytokines produced by adipose tissue macrophages, as well as dermal fibroblasts, in response to resistin and leptin, respectively, are equivalent to those that perpetuate the pathophysiology of psoriasis [reviewed in (129)].

Altogether, these observations support a role for locally produced NPY in stress-mediated responses within the skin. The future challenge is to understand how this signaling is communicating with CRH (131), POMC (46), and other local neuroendocrine stress response systems (131, 132) to affect skin physiology.

## Potential Mechanism(s) of NPY Signaling in Skin

NPY is constitutively or inducibly expressed by many cells throughout the body, and thus has pleiotropic roles in many tissues due to its wide distribution. NPY exerts its actions through interacting with its specific G protein-coupled receptors (GPCRs), which, when bound to NPY, activate G_i/o_ proteins to ultimately downregulate PKA-dependent transcription and upregulate ERK-dependent transcription (11, 31). Four of the five known receptors preferentially respond to NPY – Y1R, Y2R, Y4R, Y5R (11), although Y4R has been shown to function almost exclusively in the gastrointestinal system. Through its receptors, NPY has many known roles, including the regulation of vasoconstriction, feeding behavior, anxiety, cell proliferation, and immune cell activation and recruitment. Unfortunately, the actions that NPY has specifically in the skin are poorly understood. We can, however, use previous reports regarding the effects that NPY has on various cell populations to extrapolate how it may contribute to skin physiology and pathology.

### Keratinocytes Can Respond to NPY

Keratinocytes are the most abundant cell type in the skin, being the major component of the epidermis, and contributing to the maintenance of hair follicle structure. The main function of keratinocytes is to protect against a variety of environmental assaults, including UV radiation and microbial invasion. Although the exact effects that NPY has on keratinocytes are not yet known, Takahashi and colleagues have shown that keratinocytes are able to respond directly to NPY. In cultured human keratinocytes, 100 nM NPY suppresses up to 80% of forskolin-induced cAMP production with no effect on proliferation (133). The effect of NPY on cAMP is lost in the presence of glucocorticoids, which may be due to altered GPCR activity, and is significantly reduced at the supraphysiological concentration of 1 µM. These findings suggest that keratinocytes express at least one NPY receptor subtype, which in mouse is supported by single-cell gene expression data (19, 21).

### NPY Induces Fibroblast Proliferation and Collagen Production *via* Y1R

Fibroblasts are found in the skin’s dermis and are responsible for generating connective tissue that provides underlying structure for the epidermis and skin appendages, while also protecting the skin against, and aiding in recovery after, injury. The effects that NPY has on the skin’s fibroblasts have not yet been elucidated, however, the effects of NPY on fibroblasts from other tissues have been investigated and can give some insight into what may happen in the skin.

Both adipose-derived (3T3-L1) and skin fibroblasts have been shown to express Y1R, Y2R, and Y5R (19, 21, 134, 135). Through Y1R, 1-10nM NPY enhances primary, rat cardiac fibroblast proliferation by approximately 1.3-fold, which can be magnified with the inhibition of dipeptidyl peptidase IV (DPPIV), the enzyme that metabolizes NPY to its inactive form (136). Additionally, 10nM NPY enhances collagen production by cardiac fibroblasts *via* Y1R, which can also be magnified with DPPIV inhibition.

Dai and colleagues showed that NPY activates the fibrogenic response in hepatic pericytes. These pericytes express *NPY* and *Y1R* physiologically, and treatment with TGFβ induces pericyte activation followed by their increased synthesis and secretion of NPY (17). Moreover, treating hepatic pericytes with 10 nM NPY induces cell proliferation and migration, indicating activation of the fibrogenic response. This NPY-induced fibrogenic response is initiated by Y1R, with downstream signaling events including the phosphorylation of mammalian target of rapamycin (mTOR), 70S6K, and 4EBP1. To determine whether NPY may be involved in human hepatic diseases with fibrosis, Dai and colleagues measured the amount of NPY in the serum of healthy volunteers and patients diagnosed with fibrotic liver diseases, such as liver cirrhosis and hepatocellular carcinoma. This analysis revealed that not only is NPY elevated in the serum of patients with fibrotic liver diseases by 1.67-fold, but that the amount of NPY in serum also positively correlates with the severity of the liver disease.

### NPY Induces Proliferation and Lipid Accumulation *via* Y2R and Y5R in Adipocytes

Adipocytes, or fat cells, in the skin provide insulation, structural support, and serve as a reservoir of fatty acids that can be used for energy. More recently, adipocytes are also being recognized for their roles in the proper maintenance of overall skin and hair follicle biology through endocrine means (137). NPY acts as a pro-adipogenic peptide by inducing adipogenesis, or adipocyte maturation, and inhibiting lipolysis, or the breakdown of fats (138). However, the specific mechanisms by which NPY influences adipose tissue in different organs is still under investigation.

Immortalized murine pre-adipocytes respond to sub-picomolar concentrations up to 100 nM of NPY *via* Y1R to result in enhanced proliferation and adipogenesis, evidenced by increased lipid droplet size, lipid accumulation, and reduced expression of the pre-adipocyte gene *Dlk-1* (22, 123, 135). Surprisingly, Rosmaninho-Salgado and colleagues reported somewhat conflicting results where pre-adipocytes respond to 100 nM NPY *via* Y2R and Y5R by upregulating proliferation. Additionally, it was shown that treating mature adipocytes with 1-100 nM NPY induces lipid accumulation and upregulates the expression of mature adipocyte markers, such as C/EBPα and PPARγ, *via* Y2R and Y5R (135, 139).

### NPY Mediates Vasoconstriction in the Skin *via* Y1R and Y2R

Infusing 25-2000 pmol NPY into cutaneous veins of human skin induces robust vasoconstriction in response to cold exposure, although the ability of NPY to induce this effect is reduced or completely lost with age (140, 141). Interestingly, NPY, along with noradrenaline, is also required to prime for vasodilation in response to skin warming (142). Similarly, injecting 30-300 pmol of NPY into the microvasculature of mouse skin significantly reduces blood flow in a dose-dependent manner (28). This effect is mainly mediated by Y1R, with Y2R acting to facilitate the actions of Y1R. It is interesting to note that Chu and colleagues found no effects of NPY on edema formation or neutrophil accumulation in the skin, suggesting that NPY does not mediate microvascular permeability or neutrophil infiltration following injury in the skin. Hoffman and colleagues found that inhibition of DPPIV *via* oral administration caused elevated NPY and Y1R expression in the ductal sweat glands, endothelial cells, and arterial media within the skin of Cynomolgus monkeys, which was associated with enhanced vasoconstriction in response to NPY (143). Along with these effects, the skin of DPPIV inhibitor-treated monkeys formed blisters, and hypertrophy of dermal arterioles was also observed.

### NPY Induces Proliferation, Migration, and Angiogenesis *via* Y1R, Y2R, and Y5R in Endothelial Cells

Endothelial cells are the cells that line blood vessels and allow nutrients and signaling factors to permeate the skin to influence skin physiology. Movafagh and colleagues have shown that immortalized human endothelial cells express Y1R, Y2R, and Y5R. Additionally, these cell lines respond to NPY by upregulating proliferation, migration, and angiogenesis (144). In endothelial cells derived from dermal microvasculature (SVEC4-10’s), treatment with 10 nM and 1-10 pM NPY enhances proliferation by approximately 2-fold over regular growth medium. Likewise, in endothelial cells derived from umbilical vein (HUVECs) and SVEC4-10’s, 10nM and 1pM NPY enhances migration by approximately 2-fold. Furthermore, treating HUVEC’s and SVEC4-10’s with 0.01 pM-10nM NPY induces approximately 2-fold greater capillary tube formation, which is an early step in angiogenesis. All effects observed in response to NPY require the activity of all three NPY receptor subtypes.

### NPY Induces Proliferation *via* Y1R and Y5R in Smooth Muscle Cells

Smooth muscle cells (SMCs) are involuntary muscle cells that line the insides of organs. In the skin, SMCs control the contraction of the arrector pili muscle, which induces piloerection. Shigeri and Fujimoto showed that in the presence of insulin, treating porcine aortic smooth muscle cells with 1µM NPY for 24 hours induces approximately 1.9-fold increase in DNA synthesis and the mobilization of intracellular Ca^+2^ (145). This effect was found to be mediated by Y1R and required the presence of insulin.

Interestingly, Pons and colleagues found that rat aortic SMCs do not express technically-detectable levels of NPY receptors nor NPY itself (146), however, treatment with 100 pM NPY elevated the expression of all three NPY receptors and induced an approximately 2.5-fold increase in DNA synthesis. These effects on DNA synthesis were found to be mediated by Y1R and Y5R and could be amplified by 20-35% *via* priming cells with β-adrenergic activation or cAMP production. Additionally, Choi and colleagues showed that treating murine vascular SMCs with 20-200 nM NPY increases their uptake of acetylated low-density lipoproteins, while also increasing SMC expression of macrophage-related genes, resulting in macrophage migration toward SMCs (16).

### NPY Has Diverse Effects on Immune Cells

NPY is a neuropeptide that is expressed in almost every tissue throughout the central nervous system and the periphery, and its expression can be induced by almost every type of immune cell (20). This makes NPY an important agent of communication between the central nervous system, peripheral tissues, and the immune system. Being that the specific effects of NPY signaling are context- and receptor-specific, it should be of no surprise that NPY has diverse effects on the different immune cell populations which can be found in the skin. Lymphocytes, granulocytes, and monocytes are all found within the skin (37), although little is known about how NPY influences skin immune cells specifically. Each cell type expresses NPY receptors (25–27, 147) and thus could all respond to NPY directly.

NPY has been shown to influence human and rodent lymphocyte recruitment, proliferation, and activity *via* Y1R, Y2R, and Y5R (25, 148, 149). In primary human T cells, 10 nM NPY can induce adhesion *via* Y2R-mediated activation of β1 integrins (149). 1 pm-1µM NPY can indirectly enhance human gut lymphocyte proliferation *via* inducing IL-1β production by monocytes (23, 150). In murine T cells, picomolar to nanomolar concentrations of NPY can induce Th1 and Th2 cytokine secretion, enhance migration, and inhibit proliferation in murine lymphocytes (151–153). These effects are highly dependent on context, with the inhibitory effect of NPY changing with age and lymphocyte stimulation (153, 154). In human lymphocytes, NPY appears to also have a positive effect on lymphocyte numbers, with NPY enhancing lymphocyte proliferation in human colonic lamina propria lymphocytes (23). NPY also has effects on the Th1/Th2 balance of immune cells, promoting a Th2 polarization by upregulating IL-6 and IL-10 production in immature dendritic cells, which in turn polarize naïve T cells into Th2 cells, and increasing IL-4 production in existing Th2 cells (73, 155). Cells exposed to NPY + LPS caused a larger proportion of cells to produce IL-4 than cells exposed to LPS alone (73). This NPY induction of IL-4 secretion is also known to be independent of antigenic stimulation, which has led to speculation that neuropeptides such as NPY provide a means for the central nervous system to control the T cell response (149). Y1 receptor knockout confirms the crucial role for NPY signaling through this receptor in the production of Th2 cytokines for the local recruitment of CD4 T cells and CD11c+ antigen presenting cells in response to allergens (74). It has also been shown that NPY can interact with G coupled protein receptor 38, an orphan G protein receptor which is expressed by regulatory T cells and in several areas of the murine brain. This receptor, though dispensable for the function of regulatory T cells, has been shown through overexpression studies to lead to increased induction of FoxP3^+^ T cells *in vivo* (156, 157). NPY can also affect the proliferation of the T cells indirectly through the induction of TNF-α, as increases in NPY also cause increases in TNF-α (150, 158). More recently, it has been shown that chronic, systemic overexpression of NPY is sufficient to cause infiltration of lymphocytes, including regulatory T cells into the dermis by 22 weeks of age, with 35 week old mice retaining the same pattern of infiltration (159).

While it is unclear if there are interactions specific to resident immune cells with NPY, we previously mentioned that administration of NPY at 50 μg/kg increases the number of CD4^+^ T cells in the blood of Lewis rats (148). These results suggest that the mobilization of CD4^+^ T cells may also extend to resident memory T cells. Resident memory T cells are a population of T cells that help support the immunity to pathogens previously encountered by the body. These T cells have a different pattern of recirculation than naïve T cells, and can remain in peripheral tissue long after the previous immune challenge is gone (160, 161). Within the various types of memory T cells that have been characterized, there is a population that has been shown to preferentially migrate into the skin during inflammation that express cutaneous lymphocyte antigen (CLA) on the surface of the cell (162, 163). It was also demonstrated that this population of CLA^+^ T cells does become resident in healthy skin and provides immunosurveillance under normal conditions (164). These T cells are known to express skin specific receptors such as CCR4, CCR8, and CCR10 (165–167). There are multiple known pathways for recruitment, such as is the interaction between the skin chemokine CCL27 and CCR10 which is driven by the expression of proinflammatory cytokines (166). The majority of these cells are Th1 effector memory cells, as evidenced by the expression of IFN-γ, IL-2, and the receptor IFN-γRα being present on the cells while lacking ST2L and IFN-γRβ, which are expressed in Th2 cells (164, 168). However, in addition to this, there are distinct populations of Th2 cells and CD4^+^ CD25^+^ regulatory T cells resident in the skin as well.

Resident memory T cells are known to participate in the induction and progression of multiple skin pathologies, making them particularly interesting for investigation. Resident T cells have been shown to be sufficient to cause development of psoriasis in AGR129 mice grafted with prepsoriatic human skin (158). In active psoriasis, there is a large amount of infiltration by CD8^+^ T cells, with a large proportion of the infiltrating cells expressing integrins, CD103 and CD49a, which are unique to resident memory T cells (91). During remission of psoriasis, resident memory T cells have been shown to remain in previously affected skin and are capable of responding to cytokine stimulation long after psoriatic legions have resolved (91). Both skin homing T cells and resident memory T cells have been implicated in the pathogenesis of vitiligo. It was first shown in skin from vitiligo patients that there was a large increase in the number of CLA^+^ CD8^+^ T cells compared to skin from control patients (169). Further experiments using T cell adoptive transfer models have determined that the retention of autoantigenic resident memory T cells is required for the long-term maintenance of depigmentation in conjunction with constant expression of IFN-γ (170, 171). Finally, CD69^+^ CD103^+^ resident memory T cells have been shown to be enriched in AD skin compared to normal skin (172, 173). Studies comparing nonlesional AD skin to lesional AD skin have shown that similar types of T cells are found in both lesional and nonlesional skin, suggesting that the autoantigentic resident memory T cells may persist after remission (174). Given the association of NPY with inflammatory skin diseases, and the effect the NPY is known to have on T cell cytokine production and proliferation, further investigation on specific interactions between NPY and resident memory T cells is warranted.

NPY has also been shown to have an effect on the population of circulating B cells (148). In Lewis rats, injection of 50 μg/kg NPY caused an increase in the number of circulating IgM^+^ CD5^+^ CD11b^+^ B cells, while decreasing the number of the overall B cell population (148). This unique population of IgM^+^ CD5^+^ CD11b^+^ B cells are known in mouse as B-1 cells and are a unique subpopulation of B cells that possess unique properties in comparison to the conventional B-2 cell and regulatory B cells. One of these properties is the ability to produce antibodies for self-antigens, which is selected against in other B lymphocyte populations. This self-antigenicity serves to promote the clearing of apoptotic tissue, with many of the B-1 produced antibodies being specific for apoptotic cell debris, and it has been shown that the lack of this behavior can increase the risk of autoimmune responses due to self-reactive IgG antibodies (175, 176).

As in T and B cells, NPY can also influence NK cells, although its exact role has yet to be determined. NPY has been shown to have complex interactions with NK cells, with changes appearing to be dose-dependent, age dependent, and time dependent (177–179). In culture, 1 pM-1nM NPY acts to suppress NK cell activity, whereas intraventricular injection of 1nM NPY increases NK cell number and enhances their activity in rat (180, 181). In young mice, NPY serves to inhibit spleen leukocytes, but in aged female mice, the effect of NPY on NK cells was shown to become stimulatory in the cells of the thymus and axillary nodes (177).

Though many of the interactions discussed attribute NPY to causing immune activation and increasing the risk of autoimmunity, many of the features of NPY and its receptors likely play a role in immune homeostasis under normal conditions. Mice deficient in the Y1 receptor were found to have reduced populations of B cell, CD8^+^ T cells, and a reduced spleen size. Whereas in peripheral lymph nodes, the T cell populations were altered, having increased naïve CD4^+^ and CD8^+^ T cells, but decreased effector T cells (27). Y1 deficient T cells become hypersensitive to stimulation both *in vitro* and *in vivo*, though the production of cytokines caused from activation is unchanged (27). Changes to immune function in the absence of the Y1 receptor are not limited to lymphocyte populations. Macrophages and dendritic cells are also affected by the loss of the Y1 receptor, with macrophages showing decreased production of TNF and IL-12 following activation and dendritic cells showing decreased IL-12 production, impairment to antigen uptake, and reduced APC stimulation to T cells compared to their Y1 positive counterparts (27).

As in lymphocytes, NPY has a variety of roles in granulocyte adhesion and function *via* Y1R, Y2R, and Y5R activation. For example, *via* all three NPY receptors, 1 µM and 100 pM NPY enhances adhesion and phagocytic activity of rat peripheral blood granulocytes, respectively (182). *Via* Y1R and Y5R, 10 µM NPY similarly enhances phagocytosis in human neutrophils by stimulating reactive oxygen species production (183). Interestingly, NPY’s effects on granulocytes are dependent on the anatomical location and microenvironment – 1 nM NPY can inhibit blood granulocyte function *via* Y1R but initiate splenic granulocyte function *via* Y2R or Y5R in rats (181).

Like other immune cells, NPY also has diverse effects in monocytes, including migration, adhesion, and function. Dendritic cells and macrophages, including the skin’s Langerhans cells, can all produce NPY and express NPY receptors (16, 183–185). NPY typically plays an anti-inflammatory role in dendritic cells by promoting the production of IL-6 and IL-10 (22). NPY can also have a pro-inflammatory role in dendritic cells by inhibiting their expression of co-stimulatory molecules (74). For macrophages, NPY is a chemical attractant that promotes macrophage infiltration into tissues *via* Y1R (151). Treating macrophages with physiological concentrations of NPY (0.1-10 nM) induces their migration (186), and 20 -200n M NPY upregulates the expression and activity of matrix metalloproteinase-3 within murine bone marrow derived-macrophages (16).

In addition to the effects that NPY can exert on immune cells, NPY levels are in turn affected by the cytokines the immune cells secrete. IL-1β is produced by multiple types of immune cells as pro-IL-1β, which is then cleaved in the cytosol by caspase-1 into the active IL-1β before being secreted by the cell (187–189). NPY has been shown to induce the production of IL-1β by immune cells, with this strength of this effect being dependent on age (150, 190). IL-1β, is a proinflammatory cytokine that has been implicated in a number of autoimmune diseases both in the skin and throughout the rest of the body (102, 191). Increases in IL-1β expression and genetic variation in the *IL-1β* gene has been linked to the likelihood of developing vitiligo (102). In addition, while NPY causes an increase in the levels of IL-1β in immune cells, it has also been found that IL-1β increases the amount of NPY released by chromaffin cells (192). This process also stimulates the release of catecholamines by chromaffin cells, which have also been implicated in the skin neuroendocrine system and pathogenesis of vitiligo (34, 38, 192, 193).

### Melanocytes Do Not Directly Respond to NPY

Melanocytes are the pigment-producing cells that are located in the bulb of human and rodent hair follicles, as well as in the epidermis of human skin. Although NPY has been shown to be elevated in the depigmented skin of vitiligo patients, it is not clear whether and how NPY contributes to melanocyte destruction during vitiligo pathogenesis and progression. Due to electron microscopy findings that epidermal melanocytes are in direct contact with intraepidermal nerve endings, it has been hypothesized that NPY may negatively impact melanocytes directly (63). It has also been suggested that NPY can affect melanocytes indirectly due to NPY receptor expression by many cell populations in the skin, including fibroblasts, endothelial cells, and various immune cells (38, 63, 194). Unfortunately, these postulations have yet to be empirically evaluated in vitiliginous skin.

Toyoda and colleagues have shown that 100 nM NPY has no direct, detrimental effects on normal human melanocytes in culture (100). Likewise, treating human whole skin explants with 100 nM NPY for 72 hours has no effect on the number of melanocytes nor the amount of melanin in the epidermis. However, treating skin explants with 100 pM-1 µM NPY for 72 hours induces melanocyte degeneration and significantly reduces the number of melanosomes, the organelles in which melanin granules are stored, within melanocytes. These findings suggest that melanocytes do not respond to NPY directly, but that NPY can negatively impact melanocytes *via* paracrine signaling through other NPY receptor-expressing cell populations in the skin. In general agreement with Toyoda’s findings, a mouse model that exhibits local and chronic overexpression of NPY gene and protein in skin exhibit progressive hair graying due to melanocyte stem cell loss (101). In this case the mechanism by which NPY mediates melanocyte stem cell loss is unclear but a follow-up study indicates that chronic NPY induces inflammation and other pathogenic changes within the skin of these mice (159).

### NPY as a Skin Antimicrobial Agent

Outside of skin cells themselves, it is speculated that NPY might also interact with the skin microbiome. The surface of skin is a home to millions of beneficial and commensal microorganisms and serves as a barrier to pathogens (195, 196). Interestingly, NPY has broad antimicrobial properties against several microorganisms *in vitro*, including some that play pathogenic roles within the skin. For instance, NPY is effective at inhibiting the growth of Candida albicans, an opportunistic fungus that is elevated in the skin of individuals with chronic wounds (197, 198). It is not yet clear if these *in vitro* results reflect *in vivo* antimicrobial activity as the minimal inhibitory concentration of NPY to inhibit cell growth of Candida albicans is relatively high, 25 (56) or 240 mg/ml (197) NPY’s antimicrobial properties are also not universal; Staphylococcus aureus and Serratia marcescens, pathogenic bacteria linked to atopic dermatitis and elevated in the skin of individuals with primary immunodeficiency respectively, are both resistant to NPY (75, 197, 197, 199, 200). Nevertheless, cutaneous microbial endocrinology is a topic of growing interest and is reviewed in depth elsewhere (79, 201, 202). How NPY participates in influencing both pathogenic and commensal microbiota, either directly or indirectly through host immune regulation is worth further investigation.

## Hypothesized Roles for NPY Signaling in the Skin

**Figure 1:**
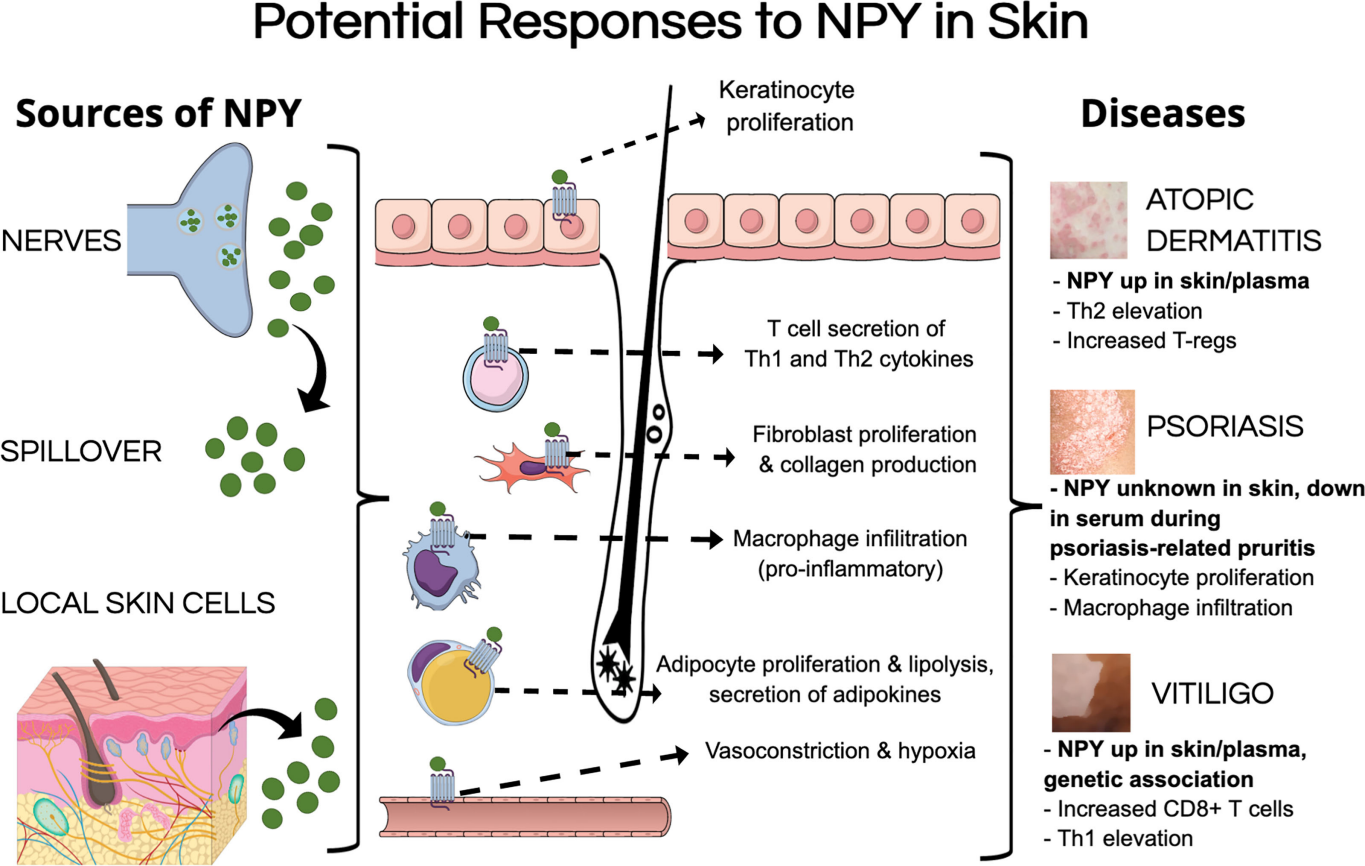
Potential mechanisms of pathological NPY signaling in the skin. NPY levels could be elevated in the skin by multiple sources, including its secretion from nerves within the skin, spillover from circulation, and secretion from different cell types in the skin. NPY could induce pathological responses in various cell types that have been shown to express NPY receptors, including keratinocytes, fibroblasts, adipocytes, and various immune cells. These pathological responses to NPY could contribute to different aspects of inflammatory skin diseases.

Despite there being little evidence of the mechanisms by which NPY can influence skin biology, we aim to extrapolate findings from other tissues regarding how NPY can influence specific cell populations to postulate how NPY may influence skin biology and contribute to pathology. Tu and colleagues showed that NPY is elevated from approximately 200 pmol in uninvolved skin to over 300 pmol in lesional skin from vitiligo patients (**Table 2**) (63). Using this elevated concentration of NPY in vitiliginous skin, we can postulate how this level of NPY in the skin can influence skin biology (**Figure 1**).

Chu and colleagues showed that up to 300 pmol of NPY induces vasoconstriction in murine skin, which reduces blood flow and oxygenation within the skin (28). Prolonged elevation of NPY to this level in the skin could result in the skin becoming a hypoxic environment, which can have detrimental effects on many cell populations. High concentrations of NPY have been shown to induce proliferation and migration in human endothelial cells (144). High concentrations of NPY also induce fibroblast proliferation, migration, and collagen production (17, 136), which could lead to fibrosis within the skin. While NPY does not seem to have any direct effects on melanocytes, Toyoda and colleagues showed that elevating NPY in the whole skin induces melanocyte degeneration (100).

Picomolar to nanomolar concentrations of NPY promote adipocyte proliferation and maturation, as well as lipid accumulation (22, 123, 135, 139). With the recent advances in our understanding of how adipocytes contribute to aspects of skin physiology, NPY-induced dysregulation of adipocyte biology within the skin could contribute to various aspects of skin pathology as has been discussed by Wong and colleagues [reviewed in (129)].

Unfortunately, presenting a generalized hypothesis about how NPY influences the skin’s immune cells is rather difficult as NPY has varying effects on different immune cell populations. Specifically, various concentrations of NPY have been shown to have both pro- and anti-inflammatory effects on lymphocytes (151–153) and monocytes (22, 74), while promoting phagocytic activity of granulocytes (181, 182, 203). Interestingly, NPY has been shown to promote macrophage migration and infiltration into tissues (16, 186), which is aligned with separate studies which show that NPY upregulates proliferation of SMCs (145, 146) and their expression of macrophage-related genes, ultimately promoting macrophage infiltration towards the NPY-activated SMCs.

Given the clinical associations between NPY expression and genetic mutations with the aforementioned inflammatory skin diseases, targeting this peptide could yield improved efficacy in combination with current therapies. For example, inhibition of NPY signaling could mitigate vasoconstriction-induced hypoxia within the skin (28), which is one of the hypothesized mechanisms that contribute to melanocyte pathology during vitiligo. Additionally, attenuation of the NPY signal in the skin could prevent NPY-induced dysregulation of fibroblasts and adipocytes (17, 22, 123, 135, 136, 139), melanocyte degeneration (100), as well as immune cell activation and trafficking to the skin (16, 151–153, 182, 186), which could diminish NPY-associated pruritus responses (200). Blocking NPY in these processes through therapeutic intervention could be promising in the treatment of AD, psoriasis, and vitiligo to enable improved qualities of life for those affected. Both peptide and non-peptide based NPY receptor-specific antagonists exist and several show potential utility in modulating specific downstream functions of NPY to improve other non-skin diseases like anxiety and alcoholism [reviewed in (11)]. The existence of these antagonists provides immediate means to further evaluate the role of NPY signaling in skin disease and may serve as the basis for therapeutics if warranted.

## Discussion

The prevalence of AD in children and adults in the United States is 12.97% (204) and 7.3% (205), respectively, and approximately 1% for individuals with vitiligo worldwide (206) Psoriasis affects up to 11% of the world’s population (207). These inflammatory skin diseases can have a significant impact on a person’s quality of life (205, 208, 209). Patients suffering from AD, psoriasis, and vitiligo report that the effect of these diseases on their physical appearance is a major source of psychological distress. Twenty-seven percent of AD patients report being bullied because of their disease (210), while psoriasis patients have been found to be significantly more likely to become excessive drinkers and smokers (211). Additionally, it has been shown that a majority of vitiligo patients suffer from social anxiety due to this disease (212). The detrimental effects that these appearance-altering skin diseases can have on those affected has led to the finding that severe anxiety and depression, as well as suicidal ideations, are significant comorbidities of inflammatory skin diseases (213). To add insult to injury, elevations in psychological stress and anxiety can also contribute to flare-ups in these diseases (69, 121, 122). Being that there are no cures for AD, psoriasis, or vitiligo, there is an immense need for more efficacious treatment strategies to combat their progression. Thus, identifying a novel contributor, like NPY, to disease pathogenesis or progression will provide a new and welcome target for clinical strategies aimed at ameliorating or reducing the severity of these diseases in these individuals.

Herein, we review what is currently known about the associations of NPY with these common inflammatory skin diseases. Along with genetic mutations that increase the susceptibility for vitiligo, increased NPY levels have been seen in the circulation of subsets of AD and vitiligo patients (60, 63, 64, 66). Additionally, the level of NPY has been shown to be elevated in depigmented lesional skin of some vitiligo patients (63). Together, these clinical findings have supported the hypothesized contributions of NPY in skin pathology (38). Unfortunately, there have yet to be empirical studies to evaluate this hypothesis, which is likely due to the lack of model with which to do so. Since we do not yet have evidence to elucidate the mechanisms by which NPY can influence skin pathology, we use here reports on the effects of NPY on various cell populations from other tissues to postulate the potential effects of NPY in the skin (see **Figure 1**). Additionally, it is possible that NPY may communicate with local neuroendocrine networks (38, 43, 46) as it does on the central level.

Despite everything that remains unknown about NPY signaling in skin, recent, novel findings suggest that we are closer than ever to elucidating mechanistic roles for this broadly expressed and multifaceted protein. Anderson et al. discovered that chronic overexpression of NPY in mouse is sufficient to act as driving factor in skin inflammation and follicular hypopigmentation (101, 159). Pathohistological and transcriptomic evaluation of these mice revealed that NPY-induced changes in skin architecture (e.g., fibrosis and hyperkeratosis), immune cell infiltration, and gene expression changes reminiscent of human inflammatory skin disease (159). This is the first evidence demonstrating that NPY signaling is capable of inducing pathological changes within the skin and highlights an animal model in which to further investigate the skin’s response to local elevations of NPY.

In summary, we provide here a concise review of what is known about the genetic associations and expression patterns of NPY in common skin diseases, the link between psychological stress and cutaneous NPY expression, and finally, we propose how NPY can contribute to skin biology. In the future, understanding NPY’s contribution to initiating and/or perpetuating skin disease, the mechanisms by which this occurs, and the connection between NPY, stress and skin are all topics which deserve greater attention. Encouraged by the discoveries of Anderson et al., investigations focused on careful monitoring of NPY expression and its source in skin disease is warranted. NPY signaling is highly amenable to therapeutic intervention as a number of NPY receptor antagonists already exist (11). In individuals where NPY signaling contributes to skin disease, NPY lends itself to being a highly druggable target that might allow for a better quality of life for those affected.

## Author Contributions

ZTA wrote the first draft of the manuscript and participated in revising the manuscript. ZTA designed the figures and tables with advice from MLH. MLH, ADD and ATS wrote sections of the initial manuscript and/or participated in revising the manuscript. All authors read and approved the final version of the revised manuscript.

## Funding

MLH, ZTA, and ADD were supported by the Department of Biology and the College of Arts and Sciences at the University of Alabama at Birmingham and by the grant ID# 67038665 from Pfizer to MLH. The authors declare that Pfizer was not involved in the study design, collection, analysis, interpretation of data, the writing of this article or the decision to submit it for publication. ATS was supported by 1R01AR073004-01A1, R01AR071189-01A1, VA merit grant (no. 1I01BX004293-01A1), R21 AI149267-01A1 and the Pfizer grant mentioned above. Fellowship support was also provided independently to ZTA (UAB Blazer Fellow, NSF LSAMP Fellow).

## Conflict of Interest

The authors declare that the research was conducted in the absence of any commercial or financial relationships that could be construed as a potential conflict of interest.

## Publisher’s Note

All claims expressed in this article are solely those of the authors and do not necessarily represent those of their affiliated organizations, or those of the publisher, the editors and the reviewers. Any product that may be evaluated in this article, or claim that may be made by its manufacturer, is not guaranteed or endorsed by the publisher.
